# Implementation of Telemedicine in a Tertiary Hospital–Based Ambulatory Practice in Detroit During the COVID-19 Pandemic: Observational Study

**DOI:** 10.2196/21327

**Published:** 2021-01-08

**Authors:** Alpana Garg, Sachin Goyal, Rohit Thati, Neelima Thati

**Affiliations:** 1 Wayne State University Detroit, MI United States; 2 Georgia State University Atlanta, GA United States

**Keywords:** telemedicine, telehealth, COVID-19, Detroit, ambulatory care, primary care, internal medicine, pandemic

## Abstract

**Background:**

The COVID-19 pandemic, caused by SARS-CoV-2, has forced the health care delivery structure to change rapidly. The pandemic has further widened the disparities in health care and exposed vulnerable populations. Health care services caring for such populations must not only continue to operate but create innovative methods of care delivery without compromising safety. We present our experience of incorporating telemedicine in our university hospital–based outpatient clinic in one of the worst-hit areas in the world.

**Objective:**

Our goal is to assess the adoption of a telemedicine service in the first month of its implementation in outpatient practice during the COVID-19 pandemic. We also want to assess the need for transitioning to telemedicine, the benefits and challenges in doing so, and ongoing solutions during the initial phase of the implementation of telemedicine services for our patients.

**Methods:**

We conducted a prospective review of clinic operations data from the first month of a telemedicine rollout in the outpatient adult ambulatory clinic from April 1, 2020, to April 30, 2020. A telemedicine visit was defined as synchronous audio-video communication between the provider and patient for clinical care longer than 5 minutes or if the video visit converted to a telephone visit after 5 minutes due to technical problems. We recorded the number of telemedicine visits scheduled, visits completed, and the time for each visit. We also noted the most frequent billing codes used based on the time spent in the patient care and the number of clinical tasks (eg, activity suggested through diagnosis or procedural code) that were addressed remotely by the physicians.

**Results:**

During the study period, we had 110 telemedicine visits scheduled, of which 94 (85.4%) visits were completed. The average duration of the video visit was 35 minutes, with the most prolonged visit lasting 120 minutes. Of 94 patients, 24 (25.54%) patients were recently discharged from the hospital, and 70 (74.46%) patients were seen for urgent care needs. There was a 50% increase from the baseline in the number of clinical tasks that were addressed by the physicians during the pandemic.

**Conclusions:**

There was a high acceptance of telemedicine services by the patients, which was evident by a high show rate during the COVID-19 pandemic in Detroit. With limited staffing, restricted outpatient work hours, a shortage of providers, and increased outpatient needs, telemedicine was successfully implemented in our practice.

## Introduction

### Background

The COVID-19 pandemic has impacted daily life and led to a rapid evolution in the structure of health care delivery. As of November 19, 2020, more than 56 million people have been infected, and 1.35 million people have died worldwide [1]. The World Health Organization declared the COVID-19 outbreak a pandemic on March 11, 2020—near the time of the first confirmed case of COVID-19 in Michigan, which became one of the major epicenters of the pandemic in the United States [2,3]. In the city of Detroit, Michigan, at the peak of the pandemic when the health care system was dealing with an unprecedented surge, outpatient clinics were taking quick and decisive steps to ensure continuity of care while keeping both patients and health care workers safe [4]. As a tertiary care institute located in the heart of Detroit, our institution cares for a predominantly African American population. Michigan is one of the ten states where prevalence of multiple chronic conditions (defined as two or more of ten diagnosed chronic conditions) is higher (30.3%) than the national average (25.7%) [5]. In addition, economic challenges and socioeconomic disparities are reflected by a high unemployment rate, high poverty rate, low health insurance coverage, and a lack of transportation, which represent significant barriers to optimal medical care [6-8]. These factors make our patient population unique and vulnerable, as evident by the higher case-fatality rate of COVID-19 in Michigan compared to other parts of the country [3,9-11].

Telemedicine involves clinical care using an electronic communication medium between two different locations [12]. Even though telemedicine existed before the pandemic, its use in health care delivery has increased tremendously during the COVID-19 pandemic [13,14]. Various health systems have already adopted remote medical care and integrated telemedicine to assist in providing care to the patients [15-18]. For our purposes, adoption is the intention, initial decision, or action to try or employ an innovation or evidence-based practice [19]. We present our experience with telemedicine in our university hospital–based ambulatory care practice during the initial phase of the COVID-19 public health emergency (PHE).

### Need for a Transition From the Outpatient Clinic to Telemedicine Clinic Model

**Figure 1:**
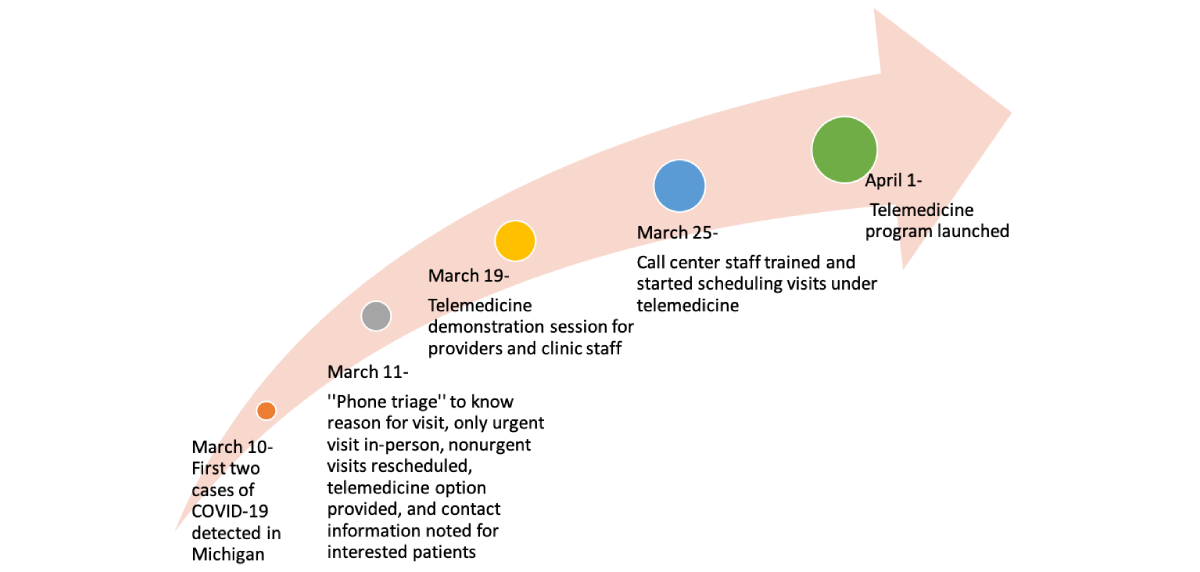
Ambulatory telemedicine clinic timeline.

Soon after the governor declared a state of emergency in Michigan, there was a sharp decline in the number of patients attending in-person visits [20]. In addition, health care providers were specially assigned tasks to “phone triage,” which meant calling all patients scheduled for an in-person visit to ask about the reason for their visits. Providers reviewed electronic medical records (EMRs), and if possible, patients’ concerns, such as medication refills or generating return to work letters, were addressed remotely. Otherwise, patients kept their in-person appointments with their health care providers. All nonurgent in-person visits were rescheduled, and patients were encouraged to stay at home to comply with safety guidelines issued by the Centers for Disease Control and Prevention [21]. With the immense reduction in the number of in-office patient visits, the number of medical assistants and clinical staff had to be reduced. Providers were being assigned to inpatient units to assist in care for the critically ill. The patient population at risk of infection had distinct needs (eg, necessary medications and medical supplies) that were in jeopardy of being unmet, which was a significant concern during the initial days of the stay-at-home guidelines. Telephone use by providers to answer patient questions and help with medication refills was done to the best that such resources would allow. In addition, certain patients were discharged from the inpatient units sooner than would be typical to decrease the risk of COVID-19 spread, and those patients needed a timely postdischarge follow-up. This caused an urgent need for a telemedicine service in our outpatient clinic. Patients were given the option of video visits, and the contact information of those patients interested in the video visits was noted. Figure 1 shows the timeline of telemedicine implementation in our practice.

### Transition From In-Person Clinic Structure to Telemedicine Clinic

#### Clinic Structure

The clinic structure before and during the COVID-19 pandemic for one half-day session is shown in Textbox 1. Before the pandemic, our ambulatory clinic was staffed with an attending physician, and all patients were seen initially by a resident physician. We had no midlevel providers in our clinic. The medical assistant and front desk staff had dedicated duties regarding patient intake and checkout, while the clinic manager took care of the overall functioning of the clinic, as established before the pandemic. In the new telemedicine setup, the medical assistant would call the patient before the telemedicine appointment to obtain additional information such as email address, confirming availability of a webcam device, the reason for visit, medication reconciliation, and pharmacy information.

**Clinic structure before and during the COVID-19 pandemic.**

**Before COVID-19**

- 8-12 resident physicians per session (half day clinic)
- 10 sessions per week
- 3 faculty members per week
- 4 medical assistants
- 2 nurses
- 2 registration staff members
- 1 clinic manager
- Fully functional registration and appointment center

**During COVID-19**

- 4 resident physicians per week
- 3-4 sessions per week
- 2 faculty members per week
- 1 medical assistant
- 1 technical support staff member
- 1 registration staff member
- 1 clinic manager
- Reduced staff at the registration and appointment center

#### Patient Scheduling

The initial patients willing to conduct a video visit were contacted as the system was being established. Additionally, all the patients who were discharged from our affiliated hospitals were instructed to follow up in the telemedicine clinic. The telemedicine providers were informed about the discharged patients via a secure email platform who would then arrange the telemedicine visit with the help of ancillary staff to ensure timely follow-up for patients. Some of these patients also included those with suspected or confirmed COVID-19.

#### Telemedicine Platform

We adopted Zoom (Zoom Video Communications Inc) and Google Hangouts Meet (Google) platforms to conduct telemedicine visits. To use these platforms for patient care, our organization entered into business associate agreements with these platforms to secure Health Insurance Portability and Accountability Act compliance and made institutional accounts for the involved physicians. This also prevented physicians from using their personal accounts for patient care to mitigate concerns regarding the sharing of personal information with patients. We circulated detailed instructions for the providers, and on-site technical staff helped with technical issues to streamline the process. A support helpline telephone number was made available for queries too. The clinic staff, including clinic manager, medical assistants, and checkout staff, were also prepared to schedule visits and helped with calling patients before their scheduled telemedicine visits.

#### Health Care Providers and Our Telemedicine Process

Physicians who had chronic medical conditions and those who were pregnant or were quarantined (due to potential exposure) but otherwise healthy were assigned to telemedicine service in addition to regular primary care physicians. All physicians were required to undergo training by completing telemedicine modules available online [22]. Providers could see their telemedicine schedule in the EMR platform and would call the patients at the appointment time either from the clinic or from the provider’s home. The resident physician and the attending physician would both join the virtual meeting, and after introductions, the resident physician was given time to assess the patient. After the resident physician assessed the patient, the patient was put on hold to discuss the assessment and plan with the attending physician. The plan was then discussed with the patient, and relevant orders for investigation and follow-up were placed in the EMR. Medical students were also able to participate in video visits. The students assigned telemedicine rotation were sent meeting links for the day so they could join the visits. To ensure adequate communication about the workflow and expectations, weekly virtual meetings with resident physicians and attending faculty were conducted.

## Methods

Prospective data were collected from the first month of telemedicine in the adult ambulatory clinic of Detroit Medical Center, Wayne State University, Detroit, Michigan. We defined a telemedicine visit as the synchronous audio-video communication between provider and patient for clinical care for 5 minutes or more. A telemedicine visit during which the call was started as an audio-video call but was switched to a telephone call after 5 minutes or more due to a technical problem was also considered a completed visit. We collected data including the number of telemedicine visits scheduled, the number of visits completed, and the most frequent billing codes used. Information about completed visits and visit durations was obtained from the provider. However, due to emergent circumstances, patient-specific information such as demographics and any protected health information was not obtained.

In our ambulatory clinic, patients were able to contact the call center to leave a message or a task for the provider (including requests directly from pharmacies for medication refill), which were directed to the internal medicine task inbox. This generated task was completed by the provider by contacting the patient via telephone or electronically, as required. The average number of tasks in the general medicine inbox were noted during the study period. Baseline information, including the number of patients scheduled per day, show rate, and the average number of tasks in the inbox for providers before the pandemic, was also obtained from the clinic manager. The numbers were recorded on Microsoft Excel spreadsheet software (Microsoft Corporation). Percentages, medians, and averages were calculated. Data were analyzed, and graphs (including box plots) were made using Python programming language software [23].

## Results

**Figure 2:**
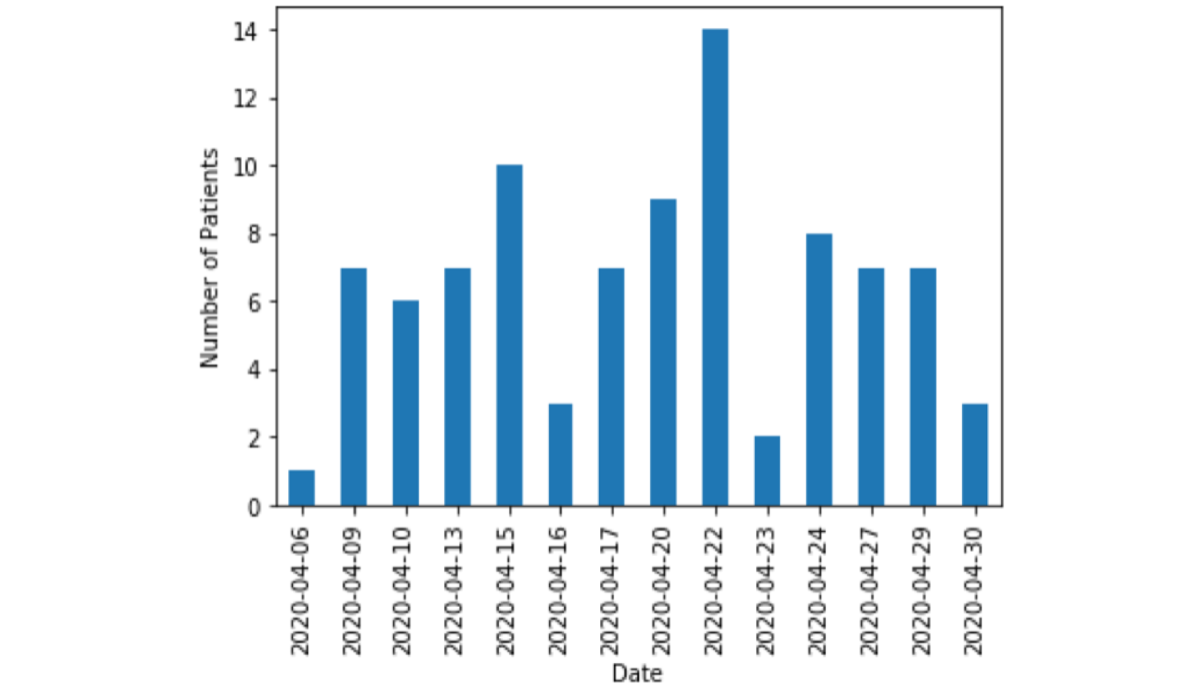
Number of patients with completed telemedicine visits over 4 weeks.

**Figure 3:**
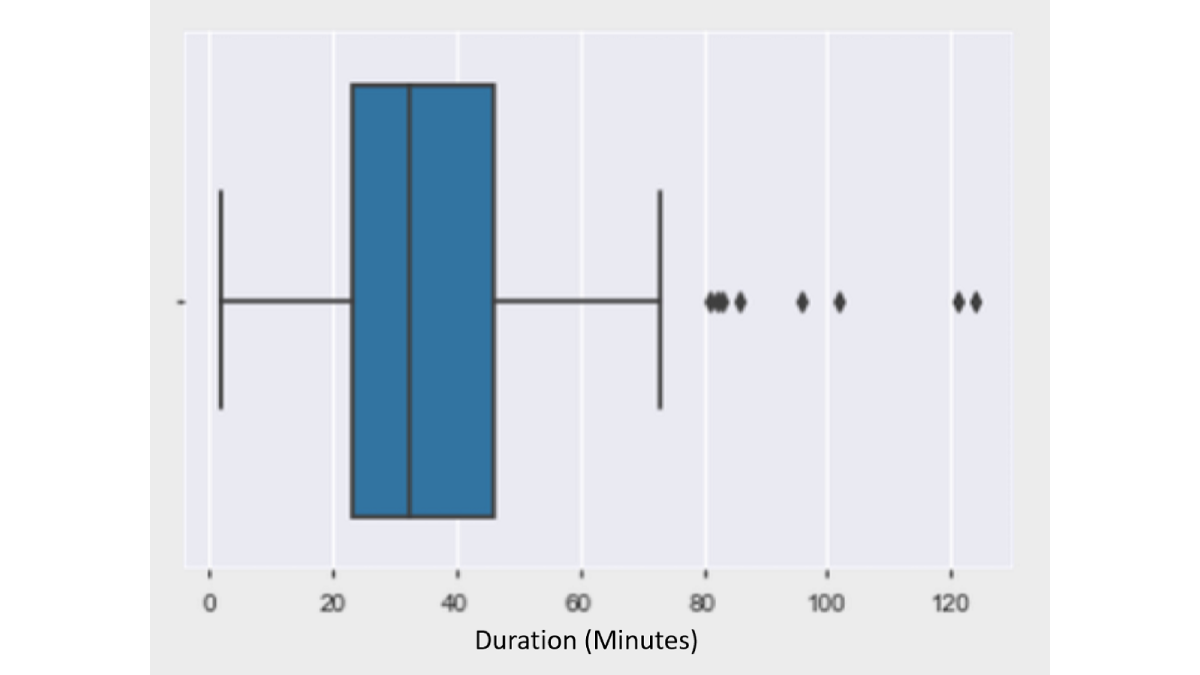
Box plot depicting duration of visits. Outliers are the values >80 minutes, and the average value is approximately 35 minutes.

**Figure 4:**
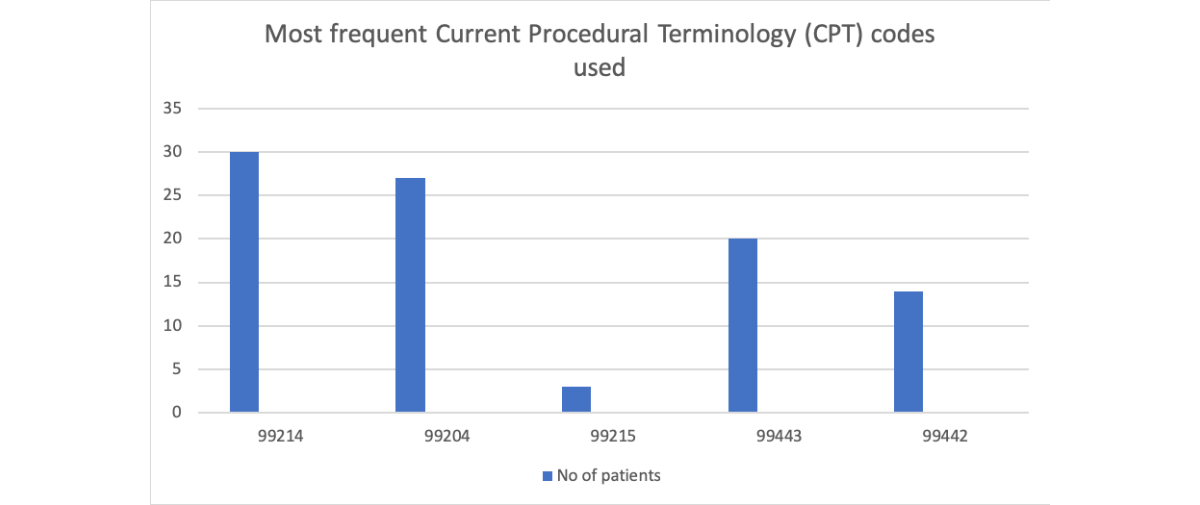
Histogram showing most common billing codes used. 99214 is an established patient office visit for 25 minutes (used with telemedicine code modifier); 99204 is a new patient office visit for 45 minutes (used with telemedicine code modifier); 99215 is an established patient office visit for 40 minutes (used with telemedicine code modifier); 99443 is a telephone evaluation and management for 21-30 minutes; 99442 is a telephone evaluation and management for 11-20 minutes.

After the initial setup, 110 patients were scheduled for telemedicine visits over 4 weeks from April 1, 2020, to April 30, 2020. Of 110 visits, 16 visits were not completed (defined in this study as when the provider was unable to contact the patient at all or the visit was shorter than 5 minutes). The total number of completed telemedicine visits distributed over 1 month is shown in Figure 2. There was no specific time pattern observed, as telemedicine scheduled visits were variable. The average length of the visit was 35 minutes (range 5-120 minutes; Figure 3). Technical challenges were encountered, such as difficulty in establishing the video call with the patient or disconnection within a few minutes. These calls were billed according to the time spent in actual patient care, excluding the time in technical challenges; calls shorter than 5 minutes were excluded. Figure 4 presents the most frequently used billing codes based on the time spent in patient care, new patient or established patient visits, and the medium (telephone or video visit). Based on visit type, 25.5% (24/94) of patients who completed telemedicine visits were recently discharged from the hospital, and 74.5% (70/94) were scheduled for telemedicine visits based on urgent needs. Most of the telemedicine visits scheduled (94/110, 85.4%) were completed. At baseline, before the pandemic, 60-70 patients were scheduled per day with a 70% show rate.

On average, 300 tasks were addressed per week by a dedicated team of resident physicians and support staff of the clinic, and included medication refills, generating referrals, producing work letters, answering queries related to COVID-19–like symptoms, and triaging. The tasks were addressed electronically or, if necessary, by calling the patient. Before the pandemic, the average number of tasks in the general medicine inbox was 200 tasks per week, which means there was a 50% increase in the number of tasks during the pandemic.

## Discussion

### Principal Findings

The adoption of telemedicine in our ambulatory practice to adjust to the unique circumstances during the COVID-19 pandemic was successful. Although the number of telemedicine visits was less than compared to in-person visits before the pandemic, the patients who had telemedicine scheduled had a high show rate of 85.4% (94/110) compared to the 70% show rate for our traditional ambulatory visits. Various factors like calling patients 2-3 days before the telemedicine visit, posthospital discharge sign-out between the providers, no requirement of transportation, the in-home convenience of a telemedicine visit, elevated levels of patient concern related to COVID-19, and reduced availability of in-person visits may have contributed to the high show rates for telemedicine. The advantages of telemedicine that we experienced are shown in Textbox 2.

**Benefits of telemedicine and telephone communications during the pandemic.**

**Continued clinical care for patients**

- Follow up on chronic medical conditions like hypertension, diabetes, asthma, congestive heart failure, and medication adjustment, and ensuring adequate number of refills

**Management of patients with possible SARS-CoV-2 infection**

- Patients with mild symptoms of COVID-19 were assessed remotely and given home quarantine instructions, testing information, and symptomatic treatment.
- Patients at home were seen through a video visit, preventing unnecessary emergency department visits with the potential to spread the disease and use critical resources.

**Decreasing hospital admission and length of stay**

- Patients who were hospitalized for conditions not related to COVID-19 and were discharged from the hospitals required a closer follow-up with primary care physicians.
- Patients with SARS-CoV-2 infection who were deemed stable for discharge but still required a follow-up after discharge (based on continued oxygen requirements or other complications) were closely monitored via a follow-up in the clinic.

**Arranging durable medical equipment**

- Increased number of requests (due to increase at-home monitoring for medical conditions) for medical equipment such as blood pressure sets, glucometers, nebulizers, and supplies for home oxygen, which were arranged in a timely manner

**Involvement of medical students and continued resident teaching and training**

- Medical students who were required to stay home during the pandemic with suspended clinical duties were still involved during the telemedicine sessions.

In the first several visits, there were technical struggles in setting up the video meeting with the patient—one visit took a total time of 120 minutes. However, we then implemented a protocol where scheduled patients were called by staff before the video visit to receive instructions to save time during the actual visit with the provider. The patients were given instructions 2-3 days before their scheduled session to allow time for downloading the applications and reviewing step-by-step instructions, which streamlined the process of setting up the video calls. If requested by the patient, written instructions were also emailed to patients. Patients and family members were informed of this secure, private platform, and they were assured that video meetings would not be recorded. Having the clinic staff call the patient before their appointment helped the providers follow the schedule and stay on time.

Through our telemedicine platform, we could follow up with patients discharged from the hospital. Discharge follow-up monitoring is important for preventing readmissions for conditions like heart failure; however, during the pandemic, it was essential to follow up with patients with confirmed or suspected COVID-19 to monitor for complications and emphasize quarantine and precautionary measures [24,25].

Weekly meetings were held with resident physicians to keep them abreast of ongoing changes and answer any specific queries. During the weekly meetings, we were able to address the challenges like limited staff available to call patients before the video visit. In addition, it was challenging for the inpatient providers to proactively set outpatient appointments through email, as initially, there was no automated system available. However, with time, staffing issues were resolved, and case managers assisted with hospital discharge follow-up telemedicine appointments. Call center staff was also urged to prioritize hospital discharge follow-up. Textbox 3 presents the challenges encountered, along with several ongoing solutions.

**Challenges during the first month of telemedicine with ongoing solutions.**

- Timely arranging for secure platform application and setting up secure links to maintain confidentiality and security for connecting patients with providers and clinic staff
  Organization created work accounts for providers and clinic staff including call center staff.
  Training sessions for providers and clinic staff
- Reaching out to patients to provide information about telemedicine and patient enrollment to the clinic for telemedicine, especially hospital discharge follow-up appointments
  Call center staff available to give options and schedule video visits for interested patients
  For hospital discharge follow-up monitoring, telemedicine order set available in electronic medical record to alert clinic staff
- Video visit mandates the presence of a smartphone or a device with a camera
  Long-term solutions to decrease economic disparity
  Assistance from family members (who can help in setting up video call) when possible
- Technical assistance required for the patients, providers, and clinic staff to set up a video call
  Patient contacted a few days before the visit by clinic staff to provide verbal and written step-by-step instructions to set up a video call before appointment
  Dedicated person to provide technical assistance to the providers and clinic staff
- Training of the resident physicians and increasing their comfort in the video visit
  Mandating completion of telemedicine modules before starting clinic
  Obtaining feedback from the residents to assess challenges encountered in the video visit and addressing them
- Establish a curriculum and ensure the teaching of resident physicians
  Dedicated teaching time to ensure learning
  Weekly ambulatory lecture including topics pertinent to telemedicine (eg, how to conduct physical exam during video visit)
- Streamline warm handoff from physician and checkout process after video visit, including methods to arrange laboratory services, referrals, and a follow-up appointment
  Dedicated clinic staff to contact the patient after video visit to explain the process to schedule laboratory tests, referrals, and follow-up appointments (to close the loop)
  Provider contacts patient to discuss laboratory results

### Limitations

This is a single institutional observational study, and hence, findings are not broadly generalizable. During the initial setup amid rapid changes, we were not able to obtain patient demographic information. Therefore, the adoption of telemedicine by various subsets of patients is unknown. We cannot determine how gender, race, ethnicity, age group, and socioeconomic disparity will affect a patient’s likelihood to schedule telemedicine visits or the impact on their ability to complete such a visit. Finally, although we ensured completion of the mandatory online telemedicine learning modules, providers learned through a rapid, on-the-job “crash course” amid a PHE, which may have impacted our results such as time required for a telemedicine visit.

### Conclusions

There was a high acceptance of telemedicine services by patients, which was evident by the high show rate during the COVID-19 pandemic in Detroit. With limited staffing, restricted outpatient work hours, a shortage of providers, and increased outpatient needs, telemedicine was successfully implemented in our practice. Quality improvement projects are needed to improve the telemedicine visit experience for both patients and providers.

## Abbreviations

EMR: electronic medical record
PHE: public health emergency
